# Eosinophils and Megakaryocytes Support the Early Growth of Murine MOPC315 Myeloma Cells in Their Bone Marrow Niches

**DOI:** 10.1371/journal.pone.0109018

**Published:** 2014-10-01

**Authors:** David Wong, Oliver Winter, Christina Hartig, Svenja Siebels, Martin Szyska, Benjamin Tiburzy, Lingzhang Meng, Upasana Kulkarni, Anke Fähnrich, Kurt Bommert, Ralf Bargou, Claudia Berek, Van Trung Chu, Bjarne Bogen, Franziska Jundt, Rudolf Armin Manz

**Affiliations:** 1 University of Lübeck, Institute for Systemic Inflammation Research, ISEF, Lübeck, Germany; 2 Charité - University Medicine Berlin, Department of Rheumatology and German Arthritis Research Center DRFZ, Berlin, Germany; 3 University of Lübeck, Institute for Anatomy, Lübeck, Germany; 4 Comprehensive Cancer Centre Mainfranken, University Hospital Würzburg, Würzburg, Germany; 5 Department of Internal Medicine II, Division of Haematology and Medical Oncology, University Hospital Würzburg, Würzburg, Germany; 6 German Arthritis Research Center (DRFZ Berlin), Berlin, Germany; 7 Centre for Immune Regulation, Institute of Immunology, University of Oslo and Oslo University Hospital, Oslo, Norway; Imperial College London, United Kingdom

## Abstract

Multiple myeloma is a bone marrow plasma cell tumor which is supported by the external growth factors APRIL and IL-6, among others. Recently, we identified eosinophils and megakaryocytes to be functional components of the micro-environmental niches of benign bone marrow plasma cells and to be important local sources of these cytokines. Here, we investigated whether eosinophils and megakaryocytes also support the growth of tumor plasma cells in the MOPC315.BM model for multiple myeloma. As it was shown for benign plasma cells and multiple myeloma cells, IL-6 and APRIL also supported MOPC315.BM cell growth in vitro, IL-5 had no effect. Depletion of eosinophils in vivo by IL-5 blockade led to a reduction of the early myeloma load. Consistent with this, myeloma growth in early stages was retarded in eosinophil-deficient ΔdblGATA-1 mice. Late myeloma stages were unaffected, possibly due to megakaryocytes compensating for the loss of eosinophils, since megakaryocytes were found to be in contact with myeloma cells in vivo and supported myeloma growth in vitro. We conclude that eosinophils and megakaryocytes in the niches for benign bone marrow plasma cells support the growth of malignant plasma cells. Further investigations are required to test whether perturbation of these niches represents a potential strategy for the treatment of multiple myeloma.

## Introduction

Multiple myeloma is a tumor of isotype-switched and somatically mutated plasma cells [1], closely related to long-lived plasma cells [2], [3]. Similar to myeloma cells, long-lived plasma preferentially home to the bone marrow [4], [5]. Although some progress has been made during the last decade with high dose chemotherapy, autologous stem cell transplantation, novel immunomodulatory drugs, such as Bortezomib, Carfilzomib, Lenalidomide, Pomalidomide, multiple myeloma is still an incurable disease with a median survival of only six years [6]–[11]. Myeloma develops slowly and progresses through three stages: (I) monoclonal gammopathy of undetermined significance (MGUS), (II) asymptomatic, or smoldering, myeloma, and (III) symptomatic myeloma. Progression from early to late stage myeloma appears to be accompanied by an accumulation of mutations leading to transformation of the original plasma cell into a more aggressive tumor cell [3], [12]. Interactions between myeloma cells and the bone marrow microenvironment are important for myeloma development and tumor progression [13], [14]. Despite current therapies not being able to efficiently eradicate multiple myeloma cells in their bone marrow environment, primary myeloma cells hardly survive in *in vitro* cell cultures and are highly susceptible to spontaneous- and chemotherapy-induced apoptosis. But resistance to apoptosis is inducible by addition of extrinsic survival factors, such as IL-6 or APRIL, among others [15]–[18]. These cytokines also represent major factors supporting the development and long-term survival of plasma cells in the bone marrow [19]–[21]. Of note - similar to what was observed for myeloma cells - the bone marrow environment seem to protect long-lived plasma cells from therapy [22]. IL-6 and APRIL are produced within specific niches that support long-lived bone marrow plasma cells [23]. Recent evidence provided by us and others demonstrates that these niches are formed by mesenchymal stromal cells together with hematopoietic cell types, such as eosinophils and megakaryocytes [24]–[26]. Evidence that normal bone marrow plasma cell populations are supported by eosinophils and megakaryocytes comes from the observations that the formation and/or persistence of long-lived bone marrow plasma cells is impaired in eosinophil deficient ΔdblGATA-1 mice, after antibody-mediated eosinophil depletion, and in c-mpl KO mice exhibiting drastically reduced megakaryocyte populations [24], [25]. Moreover, long-lived plasma cells are significantly co-localized with eosinophils and megakaryocytes [25], [27]. The idea that eosinophils support plama cells via direct cell-cell contact is supported by the finding that depletion of eosinophils results in a rapid loss of plasma cells [28]. However, in co-culture, eosinophils are able to support isolated bone marrow plasma cells merely by soluble factors, with APRIL playing a major role for this effect [25]. Hence, whether direct contact to eosinophils is required for their supportive effect on plasma cells remains to be further elucidated. Reticular stromal cells may organize the niches supporting bone marrow plasma cells through the high level production of CXCL12, which attracts precursors of long-lived plasma cells, and precursors of eosinophils and megakaryocytes[27], [29]–[32]. Interestingly, CXCR4, the chemokine receptor responsible for attracting these cells to these niches is also expressed and functionally active in multiple myeloma cells [33]–[36]. Notably, a recent paper reports that multiple myeloma cells often co-localize with eosinophils in the bone marrow and that esinophils support the proliferation of these malignant plasma cells in co-culture [37]. Hence, it seems possible that myeloma plasma cells are attracted to, and expand, in niches similar to those that support benign bone marrow plasma cells.

Here, we tested this hypothesis in the novel murine MOPC315.BM myeloma model [38]. We show that MOPC315.BM myeloma cells resemble an aggressive tumor stage, but are still supported by APRIL and IL-6. MOPC315.BM myeloma cells also expressed CXCR4, which is crucial for homing to these niches. Accordingly, mice depleted of eosinophils - the major local source of APRIL - exhibited reduced early MOPC315.BM myeloma load *in vivo*. Within the bone marrow, MOPC315.BM myeloma cells were not found in direct contact with eosinophils, suggesting that the supporting effect of eosinophils observed in this study is merely mediated by secreted factors. In contrast, MOPC315.BM myeloma cells were noticably co-localized with megakaryocytes, and co-cultured megakaryocytes supported MOPC315.BM cell growth in culture. These data indicate that even late stage myeloma cells are supported by eosinophils and megakaryocytes, i.e. hematopoietic components of the niches for benign bone marrow plasma cells, and that these niches potentially represent a novel therapeutic target for the treatment of multiple myeloma.

## Materials and Methods

### Mice and injections

BALB/c mice were purchased from Charles River Laboratories (Sulzfeld, Germany). ΔdblGATA-1 mice were bred in the animal facility of the University of Luebeck. These mice were carried mutations on GATA1 promoter which inhibit auto-regulation of GATA-1 transcriptional factor. Mice received i.v. injections of MOPC315.BM cells (1−5×10^5^ cells). Some mice received a single injection of 1 mg anti-IL-5 blocking antibodies (TRFK-5) one day before injection of MOPC315.BM cells. Challenged mice were longitudinally monitored for presence of MOPC315.BM myeloma specific anti-2,4-dinitrophenyl (DNP) IgA antibodies in sera (for details see below: ELISA). All animal experiments were approved by the respective local Committee on the Ethics of Animal Experiments of the state Schleswig-Holstein (Ministerium für Landwirtschaft, Umwelt und ländliche Räume des Landes Schleswig Holstein). All animal experiments were performed by certified personnel and all efforts were made to minimize suffering.

### Transfection and cell culture

MOPC315.BM cells were described previously [38]. This murine myeloma cell line was GFP-labeled by retroviral transfection: A retrovirus packing cell line was generated by triple-transfection of HEK 293 cells with the GFP-containing retroviral vector pMSCV and the two accessory plasmids pCAGGS-VSVg and pEQ-Pam3(-E). MOPC315.BM cells were co-cultured for 48 hours with supernatant of the retrovirus packing cell line. Subsequently, GFP-transfected MOPC315.BM cells were isolated by fluorescence-activated cell sorting. MOPC315.BM cells were grown in RPMI 1640 media (Gibco) supplemented with 10% FCS (Gibco), 0.1% Pencilllin/Streptomycin (Gibco). For investigation of growth factor requirements, IL-5, IL-6, and APRIL were added to MOPC315.BM cells in 96 well culture plates separately or in combination (100 ng/ml each). After 24 hours, Cells were washed and frequencies of living MOPC315 cells were determined by ELISPOT for DNP-specific IgA: cells were transferred to DNP-BSA (5 ug/ml) coated ELISPOT plates (cellulose ester–based) and incubated for 3 hours at 37°C, 5% CO_2_. Plates were washed, and goat anti-IgA-biotin was added (Southern Biotec). Plates were washed again and SA-HRP was added. ELISPOTs were counted after addition of ABTS.

### Co-cultures

For generation of megakaryocytes, total bone marrow cells were pre-cultured in IMDM medium containing thrombopoietin [10 ng/ml], 10% FCS and 0.1% Pencilllin/Streptomycin. After three days, megakaryocyte lineage cells were isolated by BSA-density gradient and subsequent MACS sorting for CD41+ cells. B- and T-cells were purified from spleen via MACS separation using B cell and CD4+ T Cell isolation kits (Miltenyi Biotec), respectively. MOPC315 (1,000 cells) and hematopoietic cells (10,000 cells) were co-cultured in RPMI containing 1% or 10% FCS. After two days, numbers of viable AnnexinV-, DAPI- MOPC315 GFP+, CD138+ cells were assessed by flow cytometry (using a MACS QUANT, Miltenyi Biotec).

### ELISA

MOPC315.BM myeloma load was determined by measuring myeloma specific anti-DNP IgA in serum by ELISA. Briefly: 96 well plates were coated with 10 µg/ml DNP-BSA/PBS (1 h/RT). After washing the plates three times with PBS unspecific binding was blocked (0,5% BSA/PBS; 1 h/RT). Subsequently, sera were incubated in serial dilutions for 60 min. Then, detection antibody biotinylated goat anti-mouse IgA was added (1 µg/ml) (SouthernBiotech, Birmingham, USA), and later a 3000-fold dilution of streptavidin coupled alkaline phosphatase (Roche Diagnostics GmbH, Mannheim, Germany) and finally ALP (Roche Diagnostics GmbH, Mannheim, Germany). In-between incubation steps, plates were washed three to five times. Readout was performed on a FLUOstar Omega Microplate Reader (BMG LABTECH GmbH, Ortenberg, Germany); ODs were analyzed by a (5-PL) Non-Linear Regression Curve-Fitting Model.

### Histology

Femurs were fixed in 4% PFA and then incubated in increasing concentrations of 10%, 20% and 30% sucrose over three nights. Femurs were embedded in Tissue-Tek medium (Sakura Finetek), snap- frozen in liquid nitrogen, and stored at −80°C. Tissue sections (8 µm) were prepared with a Microtome. Sections were washed in PBS and unspecific binding was blocked by preincubation with PBS/10% rat serum and anti-CD16/CD32 (clone 2.4G2; made in-house). Staining with antibodies and secondary reagents was performed for 1 hour and 30 min at room temperature, respectively. The following antibodies and staining reagents were used: goat anti-GFP-FITC (Rockland), rat anti-CD41-APC, donkey anti-Goat IgG Alexa Fluor 488 (Molecular Probes), eosinophil specific MBP 480 (Kindly donated by Claudia Berek). Sections were analyzed by confocal microscopy using a Olympus IX81 microscope.

### Flow cytometry

Bone marrow and spleen were mashed between two glass slides. Single-cell suspensions were filtered through a 70 µm cell strainer (BD Falcon) and washed twice with PBS/0.5% BSA. To block unspecific binding, cells were incubated with anti-CD16/CD32 (clone 2.4G2; made in-house) for 5 minutes on ice. Subsequently, cells were incubated with FITC labeled anti-B220 (made in-house), CD138PE (BD) and/or anti Siglec-F-PE (BD) for 20 minutes on ice. After washing, analysis was performed by flow cytometry (BD Biosciences LSRII).

### Real-Time PCR

Expression of APRIL and IL-6 was quantified in total bone marrow cells by real-time PCR. Briefly: cDNA was prepared with a Sensiscript RT kit (Qiagen), added to qPCR Master Mix (Life Technologies, Germany) and amplified using the SDS ABI 7900 system (Applied Biosystems, Darmstadt, Germany). TaqMan probes, forward and reverse primers were designed using the computer software CloneManager (version 7.01; Sci Ed Central). The optimal primmer concentrations used were 500 nm each for the forward and reverse primers and 200 nM for the TagMan probe (biomers.net, Ulm, Germany). Primer sequences: 3′ Primer sequence for IL-6: gtgcatcatcgttgttcatac; 5′ Primer sequence for IL-6: ctcccaacagacctgtctatac; 3′ Primer sequence for APRIL: aggcacggtcaggatcagaag; 5′ Primer sequence for APRIL: gcaaccagtacttaggcgtgg. The same batch of cDNA (20 µl) was used to determine the cycles of threshold (ct), and the amount of the cytokine cDNA copies were normalized to the house keeping gene MLN51.

### Statistic software

Computation and statistical analysis (Mann-Whitney U-Test or unpaired *t* test, confidence interval 95%, *P* values) were done with the GraphPad Prism 4 software.

## Results

### APRIL and IL-6 support MOPC315.BM cell growth *in vitro*

**Figure 1:**
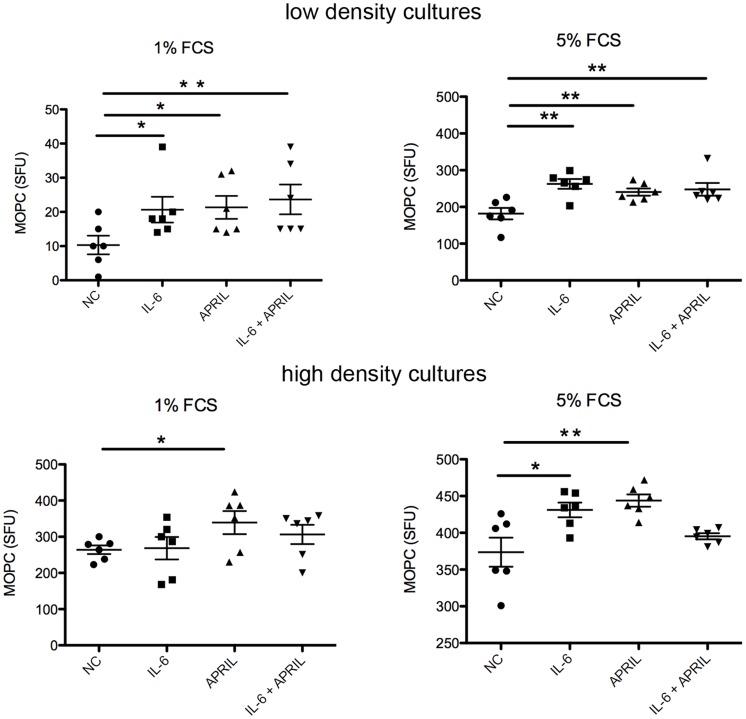
APRIL and IL-6 support MOPC315.BM cell growth *in vitro*. MOPC315.BM cells were cultured in 96 well plates containing medium supplemented with various FCS concentrations, cytokines were added alone or in combination (100 ng/ml each), as indicated. Low density cultures originally contained one thousand cells (upper graphs). High density plates were seeded with ten thousand cells. After three days of culture, living MOPC315.BM cells were quantified by a myeloma specific ELISPOT assay measuring anti-DNP-specific IgA. Three independent experiments were performed. Spot forming units (SFU) from one representative experiment are shown. Each dot represents SFUs from a single well, mean +/−SEM is shown (statistics: Mann-Whitney U test; * = P≤0.05, ** = P≤0.05).

Injection of murine MOPC315.BM cells into BALB/c mice results in tumor growth mainly in the bone marrow, suggesting that this microenvironment promotes the growth of these murine tumor plasma cells, similar to what had been observed for multiple myeloma cells in humans. Bone marrow derived IL-6 and APRIL have been shown to be major environmental-derived factors supporting the growth and drug-resistance of multiple myeloma, and also to support the survival of long-lived bone marrow plasma cells [15], [17], [23], [39]. In order to test whether these cytokines also promote the growth of MOPC315.BM cells, either 10^3^ (low density) or 10^4^ (high density) of these murine myeloma cells were cultured in 96-well plates, in medium containing either 1% or 5% FCS and with or without supplement of IL-6 and/or APRIL. After three days of culture, living MOPC315.BM cells were quantified by a myeloma specific anti-DNP ELISPOT assay. In low density cultures, the numbers of viable MOPC315.BM cells were higher in cultures containing 5% FCS compared to those with reduced 1% FCS (Figure 1). This difference of myeloma cell growth in cultures containing different FCS concentrations was much less pronounced in high density cultures. In low density cultures, both the addition of IL-6 or APRIL led to increased yield of myeloma cells three days later, regardless whether the cultures contained 1% or 5% FCS. Similar results were observed at days one and fife (Figure S1). Addition of IL-6 and APRIL together did not result in increased myeloma growth compared to supplement with one cytokine alone. In contrast, although IL-6 and APRIL supported myeloma growth in some high density cultures, this effect was not consistent. These results suggest that high myeloma cell density can substitute for external growth factors, most likely by provision of cell-cell contact or production of autocrine growth factors. This idea is consistent with the findings that human myeloma cells and under certain conditions even normal plasma cells can produce autocrine growths factors, such as IL-6 and APRIL [40], [41]. Together, these results indicate that MOPC315.BM cells are supported by IL-6 and APRIL, as have been previously reported for multiple myeloma [15], [42]–[44]. However, MOPC315.BM dependency on these external growth factors is lost when MOPC315.BM cells have reached high cell densities.

### MOPC315.BM cells represent an aggressive tumor stage

**Figure 2:**
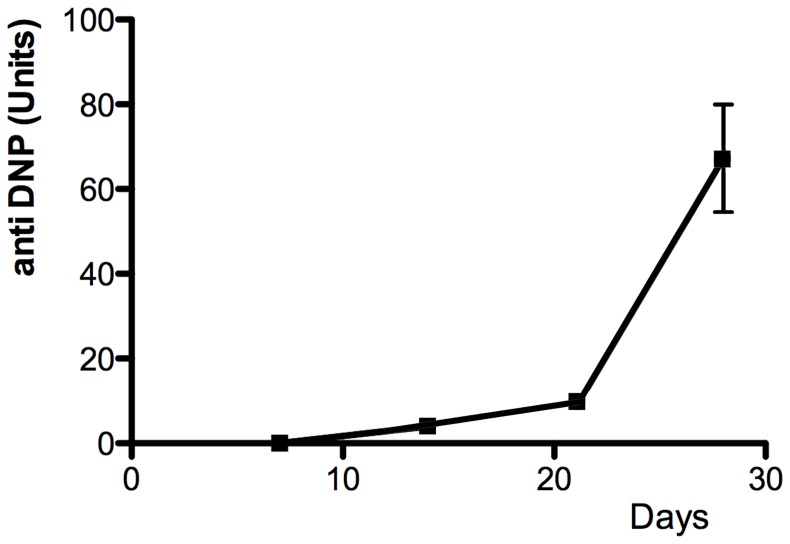
Kinetics of MOPC315.BM myeloma load. MOPC315.BM cells were i.v. injected into BALB/c mice (500,000 cells/mouse). At the time points indicated M315 myeloma protein specific for the hapten DNP was measured in sera by ELISA.

In order to address MOPC315.BM cell growth *in vivo*, MOPC315.BM cells were injected i.v. into mice. Within five weeks, mice that had received 500000 MOPC315.BM cells developed a high myeloma tumor load as indicated by serial measurement of myeloma protein in sera (Figure 2), finally resulting in paraplegic disease due to spinal involvement. In accordance with what has been published earlier by Hofgaard and colleagues [38], after three weeks most MOPC315.BM cells were found in the bone marrow, while minor populations were present in the liver and spleen (data not shown). Both, rapid myeloma disease development and autocrine growth in high density cultures suggest that the MOPC315.BM model may represent an aggressive myeloma stage. Preferential homing of MOPC315.BM cells to the bone marrow, as well their myeloma-related growth factor requirements at low cell densities *in vitro*, indicate that MOPC315.BM represents a suitable model for analyzing the interaction between late stage myeloma cells and their bone marrow environment.

### Early MOPC315.BM growth *in vivo* is reduced after eosinophil depletion and in eosinophil deficient mice

**Figure 3:**
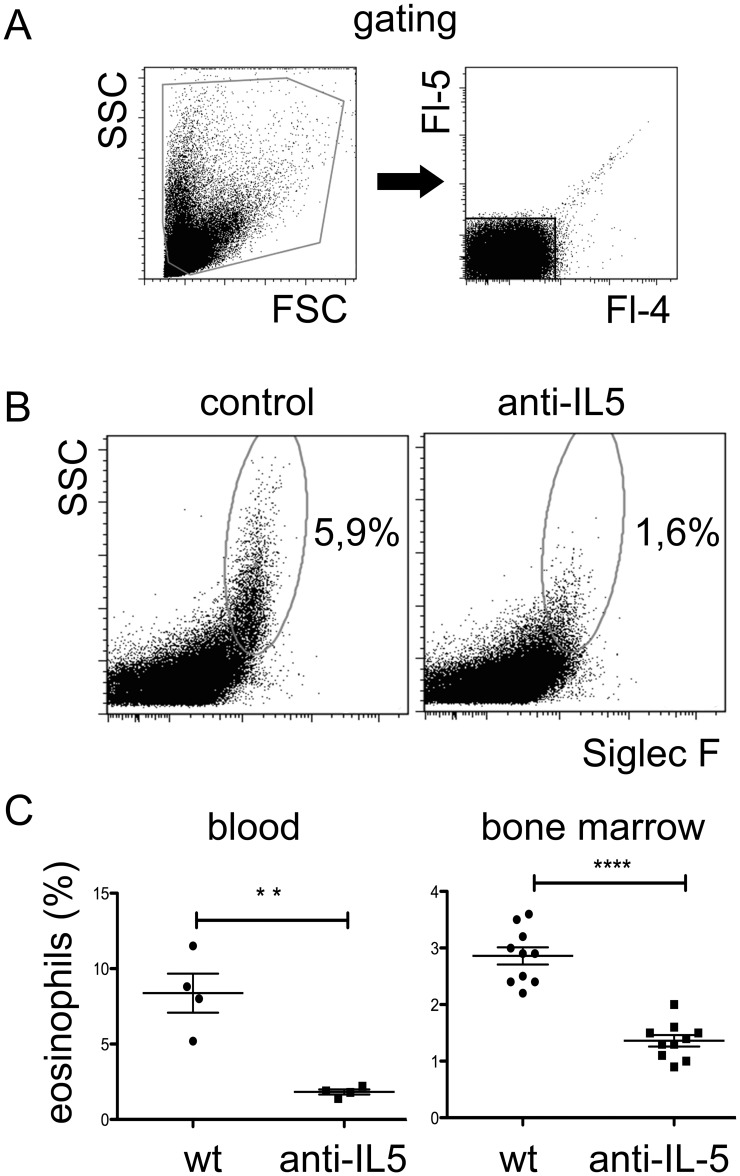
Anti-IL-5 treatment efficiently depletes eosinophils from blood and bone marrow. Mice received single injections of one mg/ml anti-IL-5 blocking antibody. Eosinophils were quantified by flow cytometry. (A) Gating strategy: cells were electronically gated through a forward scatter (FSC)/side scatter (SSC) gate followed by exclusion of autofluorescent cells by using two irrelevant fluorescence parameters (Fl-4 and Fl-5). In addition, doublets were excluded according to light forward scatter width (not shown). (B) Eosinophils are identified by Siglec-F expression and SSC. Representative data are shown from blood of PBS treated control mice and mice receiving anti-IL-5 blocking antibody, as indicated. (C) Average eosinophil frequencies in blood and bone marrow one week after injection of anti-IL-5 or control antibodies. Each dot represents the eosinophil frequency of an individual mouse. Mean +/− SEM are shown (statistics: Mann-Whitney U test; ** = P≤0.05, *** = P≤0.001).

**Figure 4:**
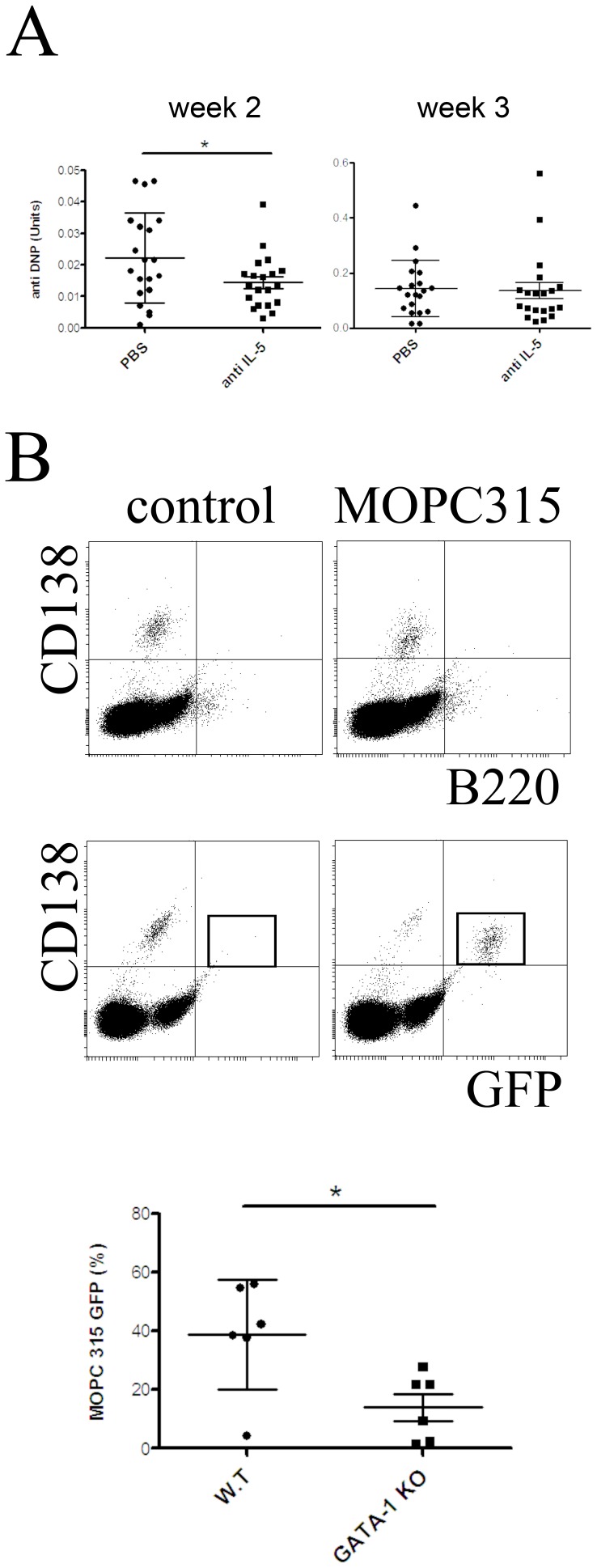
Eosinopenia impairs MOPC315.BM myeloma growth *in vivo*. (A) In order to induce eosinopenia mice were treated with anti-IL-5 blocking antibody. One week later, 5×10^5^ MOPC315.BM cells were injected and myeloma load was determined by quantification of myeloma-specific serum protein (anti-DNP) by ELISA. Each dot represents myeloma specific protein from one mouse at week two after myeloma cell injection (statistics: unpaired t-test; * = P≤0.05). (B) Top: Example of flow cytometric analysis of CD138, B220 and GFP expression in bone marrow of mice that four weeks earlier received either MOPC315.BM transfected with eGFP, or PBS (control). MOPC315 cells were identified according their high level expression of GFP (as indicated by quadrant). Bottom: eGFP-labeled MOPC315.BM cells were injected into eosinophil deficient ΔdblGATA-1 mice and wild type mice, both on a BALB/c background. Two weeks after injection, eGFP-labeled myeloma cells were quantified in the bone marrow by flow cytometry. Each dot represents MOPC315.BM counts in one mouse. Data shown in part A and B represent data from two independent experiments. Mean +/− SD are shown (statistics: Mann-Whitney U test; * = P≤0.05).

Eosinophils are a prominent source of APRIL in the bone marrow and have been identified as a major functional component of the niches for long-lived plasma cells [25], [26]. Antibodies blocking the eosinophil growth factor IL-5 have been shown to reduce eosinophil numbers in blood and tissues of mice [45], and several therapeutic anti-human IL-5 monoclonal antibodies that are able to reduce eosinophil counts in patients are under development [46]. The finding that APRIL supports MOPC315.BM growth *in vitro* suggests that prominent APRIL producing cells such as eosinophils could also support MOPC315.BM growth *in vivo*. In order to test this hypothesis, mice were treated with a single high dose of one mg anti-mouse IL-5 blocking antibody. Already one week after treatment eosinophil numbers were about 90% and 70% reduced in peripheral blood and bone marrow, respectively (Figure 3). Eosinopenia was maintained for at least four weeks (Figure S2). In accordance with the idea that eaosinophils resemble a major source of APRIL, anti-IL-5 treatment reduced the expression of this cytokine within the bone marrow (Figure S2). In order to study the effect of eosinophil depletion on MOPC315.BM cell growth *in vivo*, MOPC315.BM cells were injected into BALB/c mice that have been treated one week earlier with anti-IL-5. Two weeks after MOPC315.BM injection, the myeloma load was approximately 30% reduced in anti-IL-5 treated mice, as measured by myeloma-specific anti-DNP protein in serum (Figure 4). However, at later time points no significant differences between the treated and untreated groups were detected. Of note, IL-5 did not support the secretion of myeloma specific anti-DNP antibodies (Figure S3). Instead, high concentrations of IL-5 even seem to decrease the secretion of anti-DNP in culture. Hence, excluding the possibility that the decrease of myeloma specific protein in anti-IL-5 treated mice was due to suppression of antibody secretion. Together, these results suggests that IL-5 blockade can suppress the early, but not the late MOPC315.BM growth phase *in vivo*. This is consistent with the finding that MOPC315.BM cells became independent of external growth factors at high cell densities, which was also observed in culture. Of note, IL-5 has not been implicated as a growth factor for myeloma cells and does not support the growth of MOPC315.BM cells *in vitro* (Figure S4). These results suggest that reduction of myeloma load after anti-IL-5 injection is due to the depletion of eosinophils.

When the number of injected MOPC315.BM cells was reduced to 100000 per mouse, the myeloma load increased at a lower rate and the variation of the myeloma load within the groups became very high. Four weeks after injection, myeloma specific anti-DNP was present in all mice of the control group (10/10), but in only 70% (7/10) of IL-5 treated mice (Table 1). Consistent with the effect of anti-IL-5 blocking antibodies on the myeloma load after injection of high numbers of MOPC315.BM cells, eosinophil-depletion showed its capacity to reduce early myeloma growths.

**Table 1:** Impact of anti-IL-5 treatment on incidence of early myeloma development.

	no of mice exhibiting nodetectable myeloma load	no of mice with lowmyeloma load	no of mice with intermediatemyeloma load	no of mice with highmyeloma load
control group	0	3	4	3
anti-Il-5 treated group	3	3	3	1

MOPC315 BM. cells were injected at low doses (one hundred thousand per mouse). Anti-IL-5 blocking antibody or PBS (control) was injected one day before injection of the myeloma cells. Myeloma specific anti-DNP in serum was quantified as a measure for the myeloma load. Data shown represent myeloma load at day 28 after injection of MOPC315 BM. Cells. Detection limit = 2 units anti-DNP; low myeloma load = between 2 and 50 units anti-DNP; intermediate myeloma load = between 50 and 100 units anti-DNP; high myeloma load = above 100 units anti-DNP.

In order to probe this idea further MOPC315.BM cells were injected into ΔdblGATA-1 deficient mice. These mice exhibit an eosinophil deficiency but otherwise a normal hematopoiesis. Most likely as a consequence of the absence of eosinophils, APRIL levels are severely reduced within the bone marrow of such mice [25]. Before injection, MOPC315.BM cells were retrovirally transfected with eGFP, allowing for the identification by flow cytometry. In culture, transfected MOPC315.BM cells were supported by APRIL and IL-6 similar to non-transfected cells (data not shown). When mice were analyzed two weeks after injection, ΔdblGATA-1 mice showed an approximately 60% reduction in the frequencies of MOPC315.BM cells in the bone marrow compared to control mice (Figure 4). Noteworthy, eGFP-transfected MOPC315.BM cells homed not only to the bone marrow, but to an increased extent also to other tissues. Similar to what have been reported for “normal” plasma cells, MOPC315.BM loads were reduced in the bone marrow but not in other tissues of ΔdblGATA-1 deficient mice. These results suggest that eosinopenia specifically impairs the myeloma supporting capacity of the niches in bone marrow. Together, these data indicate that depletion of eosinophils - either by anti-IL-5 or in genetically modified mice - impairs the growth of MOPC315.BM myeloma cells in the bone marrow during early myeloma development.

### Megakaryocytes support MOPC315.BM myeloma cell growth in co-culture

**Figure 5:**
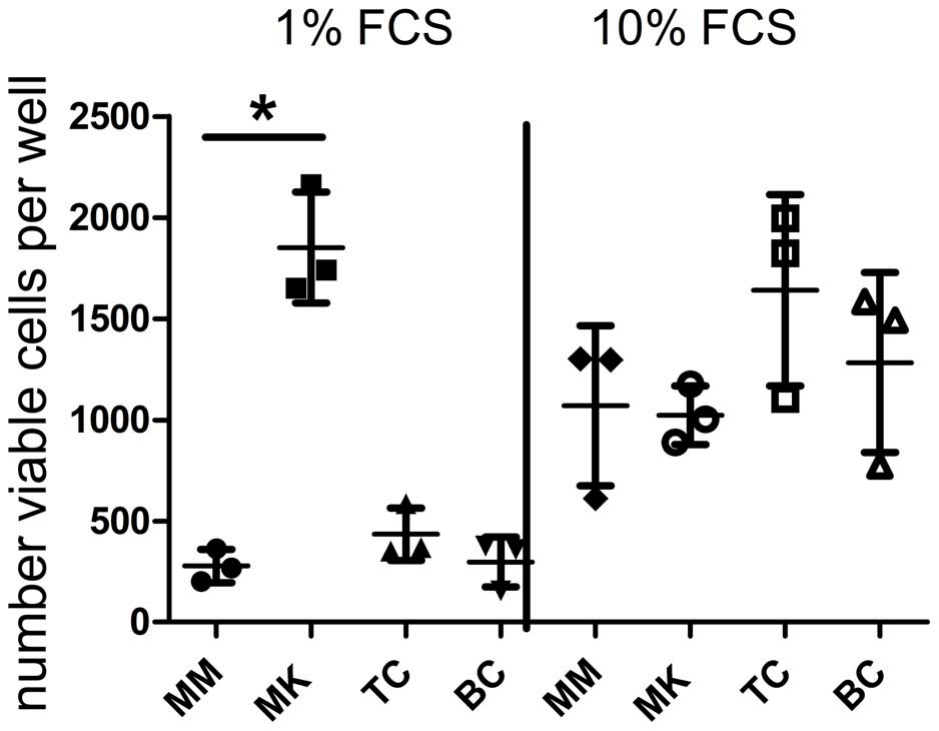
MOPC315.BM myeloma cell growth is supported by co-cultured megakaryocytes. MOPC315.BM cells were cultured at low densities (1000 cells/well) in 96 well plates alone (MM) or together with *in vitro*-generated megakaryocytes (MK), CD4+ T cells (TC) or IgD+ B cells (BC) in medium supplemented with either 1% or 10% FCS, as indicated. Ratio of myeloma cells to feeder cells was 1∶10. After two days of culture the numbers of viable (AnnexinV-, DAPI-) MOPC315.BM cells (GFP+, CD138+) were quantified by flow cytometry. Each dot represents myeloma cell counts from a single well. Data are representative for four independent experiments. Mean +/− SEM are shown (statistics: Mann-Whitney U test; * = P≤0.05).

Megakaryocytes represent another hematopoietic cell type that contributes to the niches supporting benign long-lived bone marrow plasma cells [24], [27]. Megakaryocytes constitutively produce IL-6, a major growth factor for both benign and malignant plasma cells. Unfortunately, the MOPC315.BM model is derived from BALB/c mice and is not compatible with existing mouse models exhibiting specifically and massively reduced megakaryopoiesis, such as c-mpl KO mice. Therefore, the potential supportive role of megakaryocytes for MOPC315.BM myeloma cell growth was investigated in an *in vitro* co-culture setting. Myeloma cells were seeded in low density cultures as described above, together with various cell types in ratios of 10 to 1 (feeder cell to myeloma cell). In cultures supplemented with 1% FCS, *in vitro*-generated and purified CD41+ megakaryocyte lineage cells resulted in an approximately 2 to 6-fold increase in myeloma cell numbers compared to myeloma cells cultured without feeder cells (Figure. 5). B cells or CD4+ T cells had no effect. In the presence of 10% FCS, that boosted growth of MOPC315.BM, megakaryocyte lineage cells had no additional effect on myeloma cell growth. These data demonstrate that megakaryocytes exhibit a supportive effect on myeloma cell growth. These observations are in accordance with the idea that hematopoietic components of the niches for benign plasma cells can also support the growth of myeloma cells, although this effect might be relevant only under suboptimal conditions, i.e. low cell density culture or low frequency among total bone marrow cells during the early growth phase *in vivo*.

### MOPC315.BM myeloma cells co-localize with megakaryocytes in the bone marrow

The chemokine receptor CXCR4 is crucial for the homing of long-lived plasma cells to the bone marrow niches [47]–[49]. The CXCL12/CXCR4 axis can also recruit precursors of eosinophils and megakaryocytes to the CXCL12-producing stromal cells that seem to form the scaffold of the plasma cell supporting bone marrow niche [27]. Similarly, CXCR4 has also been implicated in the bone marrow specific homing capacities of multiple myeloma cells [50]. Flow cytometric analysis revealed that this chemokine receptor is also expressed on MOPC315.BM cells (data not shown). In order to investigate whether indeed MOPC315.BM cells preferentially home into the same micro-environmental niches that support the survival of long-lived plasma cells, the cellular environments of eGFP-labeled MOPC315.BM cells were analyzed by confocal microscopy. During later stages of myeloma growth – typically starting from three to four weeks after injection - MOPC315.BM cells occupied a significant proportion of the whole bone marrow, which made co-localization studies difficult (data not shown). At earlier time points myeloma cells were often hard to detect, while samples exhibiting intermediate myeloma load were very rare. These observations suggest that the early phase is characterized by only a few myeloma cells in bone marrow that do not significantly expand for approximately two-to-three weeks. This phase seems to be followed by a short phase of rapid expansion of myeloma cells. However, this issue needs further investigation.

**Figure 6:**
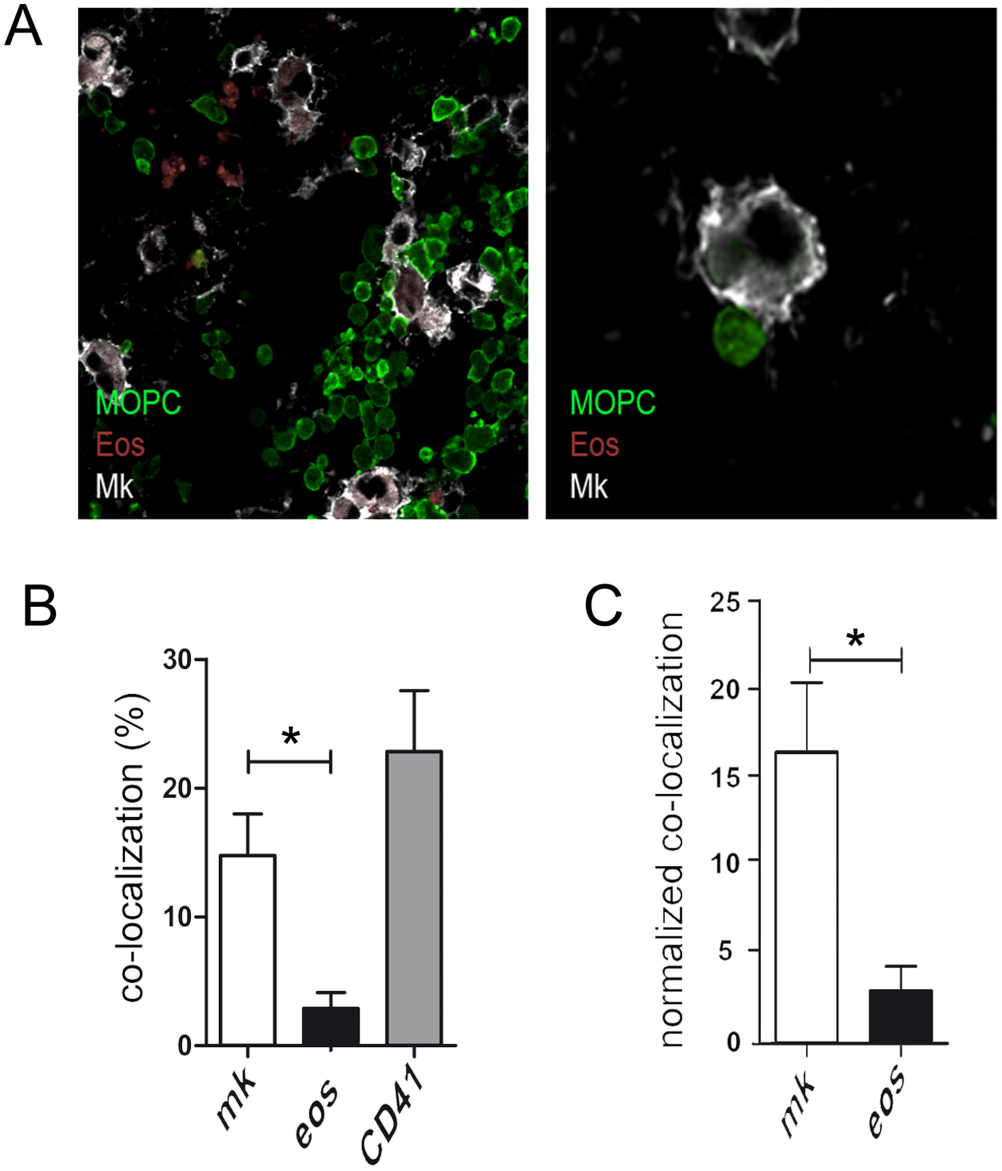
MOPC315.BM myeloma cells co-localize with megakaryocytes *in vivo*. Cryosections of murine bone marrow (femur) from mice with moderate load of eGFP-labelled MOPC315 BM cells. (A) Sections were stained for eosinophils (MBP, red) megakaryocytes (CD41, white). MOPC315 BM cells were identified by eGFP (green). Analysis was performed with a Olympus IX81 confocal microscope using a 20x oil objective and processed with Olympus microscope and Adobe Photoshop software. Left: representative picture from a femur exhibiting intermediate myeloma load. Right: myeloma cells often make direct contact to megakaryocytes. (B) In femurs of three mice a total of 1244 MOPC315.BM myeloma cells were screened for their contacts to MBP+ eosinophils and CD41+ megakaryocytes, respectively. Contacts to CD41+ “particles” which could not clearly morphologically identified as a megakaryocyte were counted separately. Left: Bars show the respective co-localization frequencies for myeloma cells Right: Contact frequencies normalized on the total cell surface areas of megakaryocytes and eosinophils, respectively. Normalized data presented as contacts of one hundred myeloma cell to one square micrometer of the respective niche cell. One experiment was performed. Mean +/− SD are shown (statistics: Mann-Whitney U test; * = P≤0.05).

In samples of femurs exhibiting an intermediate myeloma load where individual myeloma cells and small clusters of myeloma cells were detectable, co-localization between myeloma cells, MBP+ eosinophils and CD41+ megakaryocyte lineage cells was studied. Of a total of more than 1200 eGFP-labeled MOPC315.BM cells from femurs of three mice analyzed, approximately 2.9% of myeloma cells were co-localized with eosinophils (Figure 6). This is much below the 60% of long-lived plasma cells that has been previously reported to co-localize with eosinophils [25]. The low co-localization between MOPC315.BM cells and eosinophils indicates that the observed eosinophil-mediated support of MOPC315.BM cells may not require direct cell-cell contact. This finding is in accordance with the observations that eosinophil deficiency in ΔdblGATA-1 mice results in a severe reduction of APRIL expression and that eosinophil support of long-lived plasma cells and myeloma plasma cells is mediated by soluble factors [25], [37]. Contacts between MOPC315.BM cells and megakaryocytes were approximately 12-times more frequent than contacts between MOPC315.BM cells and eosinophils (Figure 6). These contact frequencies were normalized for the total cell surface areas of megakaryocytes and eosinophils which provide a measure for their relative contact probabilities, as described earlier [24]. The normalized contact frequencies per cell surface area were approximately 10-times higher for MOPC315.BM cell contacts to megakaryocytes compared to contacts to eosinophils. This result shows that the contacts between MOPC315.BM cells and megakaryocytes are much more frequent than expected in a setting where both cell types are randomly distributed, possibly suggesting that MOPC315.BM cells and megakaryocytes are attracted to the same niche. Of note, another 22.6% of MOPC315.BM cells were found in contact with “particles” that stained for the megakaryocyte lineage marker CD41, but could not be clearly identified as megakaryocytes on the basis of morphology. Whether these contacts indicate previous interaction to megakaryocytes needs to be elucidated.

## Discussion

Multiple myeloma is a so far incurable tumor of bone marrow plasma cells. Here, we report that murine MOPC315.BM myeloma cells have similar growth factor requirements and homing capacities as multiple myeloma cells and therefore represent a suitable model for studying the interaction of myeloma cells with the micro-environmental niches for normal plasma cells. The data presented indicate that the hematopoietic components of these niches – eosinophils and megakaryocytes –support also the growth of MOPC315.BM myeloma cells.

Notably, IL-5 blockade-mediated eosinophil depletion reduced the early myeloma load, providing a proof of principle that therapeutic targeting of hematopoietic components of plasma cell niches represents a novel strategy for suppressing multiple myeloma. Although the therapeutic effect of eosinophil depletion in the MOPC315.BM myeloma model is not pronounced, it is still remarkable because MOPC315.BM myeloma cells resemble a late and aggressive tumor stage.

When cultured in higher densities in medium supplemented by high FCS concentrations, MOPC315.BM myeloma cells did not require any external stimulation by APRIL or IL-6. Such a growth behavior is similar to that observed for myeloma cell lines resembling late stage tumors but differs from that of primary myeloma cells, which exhibit stronger dependency on external stimulation [51]. Primary multiple myeloma cells derived from patients might be more closely related to untransformed benign bone marrow plasma cells than MOPC315.BM myeloma cells. In accordance with this, Wong and colleagues recently described that multiple myeloma cells often co-localize with eosinophils *in vivo*, similar to what has been previously described for benign bone marrow plasma cells [37]. The same study also showed that eosinophils support the growth of multiple myeloma cells *in vitro* via secretion of soluble factors and suggested an IL-6 independent mechanism of supporting of myeloma cell growth. The findings presented in our study suggest that decreased APRIL and IL-6 production likely contributes to the reduced MOPC315 growth observed in ΔdblGATA-1 mice and after IL-5 treatment. However, suppression of other eosinophil derived myeloma growth factors might be also involved. Whether eosinophils and megakaryocytes express other prominent myeloma growth factors such as insulin-like growth factor type 1 (IGF-1) [52], [53], needs to be elucidated. Clearly, individual components of the niche can produce multiple important myeloma growth factors. Therefore, we propose that targeting of niche cells might be a more efficient strategy to suppress myeloma growth than blockade of individual growths factors.

Together, multiple myeloma cells and cells of the MOPC315.BM model are supported by eosinophils-derived factors but only the former are often directly co-localized to eosinophils. Hence, again suggesting that MOPC315.BM cells resemble a late stage tumor and that most multiple myeloma – particularly during the early stage of disease - are likely to be more affected by eosinophil depletion than what we observed in this late stage tumor model. Therefore, we conclude that treatment of early stage multiple myeloma with anti-IL-5 blocking reagents is worth a trial in patients. Multiple anti-human IL-5 blocking reagents are under development for the treatment of eosinophilic conditions and initial studies indicate that these biologicals are well tolerated [54], [55].

At least in culture, addition of APRIL and IL-6 together did not support myeloma growth above that of the single factors added alone, suggesting that the two cytokines exhibit redundant effects for supporting myeloma growth. Other factors such as IGF-1 are also important for myeloma growth *in vivo* [52], [53]. These observations may explain why IL-6 depletion did not turn out to be very effective for the treatment of myeloma patients, and suggests that targeting of the niche cells that simultaneously provide several myeloma supporting factors could be more effective.

Whether eosinophils represent the most important niche cells supporting myeloma growth needs to be elucidated. The observations that megakaryocytes represent an important source of IL-6 [24], support MOPC315.BM myeloma cells in co-culture, and are co-localized with MOPC315.BM myeloma cells *in vivo,* suggest that megakaryocytes can substitute for the supportive effect of eosinophils on myeloma growth in the bone marrow. Therefore, an optimal strategy for blocking the supportive effect of plasma cell niches on myeloma growth may require combined targeting of multiple niche components, such as eosinophils and megakaryocytes at the same time.

## Supporting Information

Figure S1 **Kinetics of APRIL mediated support of MOPC315.BM cell growth in vitro.** MOPC315.BM cells were cultured in 96 well plates containing medium supplemented with 5% FCS concentrations, as described in Figure 1. After one, three and fife days of culture, living MOPC315.BM cells were quantified by a myeloma specific ELISPOT assay measuring anti-DNP-specific IgA. One experiment was performed. SFU = Spot forming units (SFU). Each dot represents SFUs from a single well, mean +/−SEMSD is shown (statistics: Mann-Whitney U test; ** = P≤0.05).
(TIF)

Figure S2 **Eosinophil depletion by anti-IL-5.** Mice received a single injection of 1 mg anti IL-5 blocking antibody (TRFK-5) or PBS. (A) Subsequently, eosinophil frequencies were analysed in blood by flow cytometry, at the time points indicated. Each spot resembles the average of three individuals. (B) At day seven after injection of anti-IL-5 antibodies, expression of APRIL mRNA and IL-6 mRNA by total bone marrow cells were quantified by RT-PCR. RU = relative units. Statistics: Mann-Whitney U Test; * = P≤0.05. Data are representative of one experiment with three mice per group. Mean +/− SD are shown.
(TIF)

Figure S3 **Effect of IL-5 on the production of myeloma specific protein.** MOPC315.BM cells were cultured in 96 well plates containing medium supplemented with 1% FCS concentrations, as described in the methods section. IL-5 was added every other day. Myeloma specific anti-DNP IgA in supernatants from days three, fife and seven was measured by ELISA as described in the methods section. Each dot represents a single well. Statistics: Mann-Whitney U Test; * = P≤0.05.
(TIF)

Figure S4 **IL-5 does not support the growth of MOPC315.BM cells.** MOPC315.BM cells were cultured in 96 well plates in high density cultures as described in Figure 1, with medium supplemented with 5% FCS. IL-5 was added at 100 ng/ml, control wells received no cytokine (NC). After three days of culture, living MOPC315.BM cells were quantified by a myeloma specific ELISPOT assay measuring anti-DNP-specific IgA. Each dot represents the number of spot forming units (SFU) from a single well. Differences were not significant, as determined by an unpaired t-test.
(TIF)
