# Supplementary figures and images for: Eosinophils and Megakaryocytes Support the Early Growth of Murine MOPC315 Myeloma Cells in Their Bone Marrow Niches
Source: PLoS One. 2014 Oct 1;9(10):e109018. doi: 10.1371/journal.pone.0109018 (PMC4182881; doi:10.1371/journal.pone.0109018)

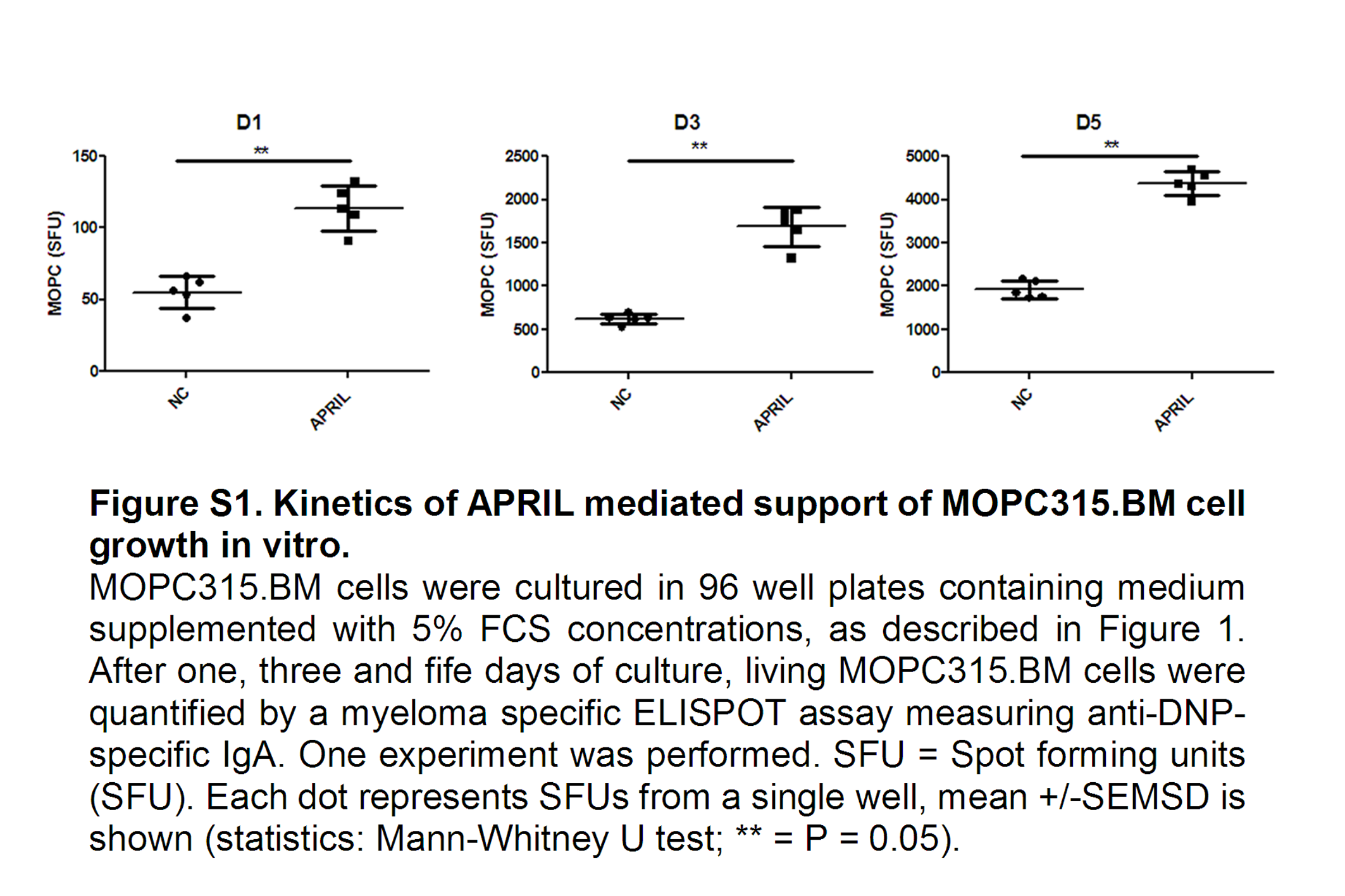

Supplement: Figure S1 — Kinetics of APRIL mediated support of MOPC315.BM cell growth in vitro. MOPC315.BM cells were cultured in 96 well plates containing medium supplemented with 5% FCS concentrations, as described in Figure 1. After one, three and fife days of culture, living MOPC315.BM cells were quantified by a myeloma specific ELISPOT assay measuring anti-DNP-specific IgA. One experiment was performed. SFU = Spot forming units (SFU). Each dot represents SFUs from a single well, mean +/−SEMSD is shown (statistics: Mann-Whitney U test; ** = P≤0.05). (TIF) [file pone.0109018.s001.tif]

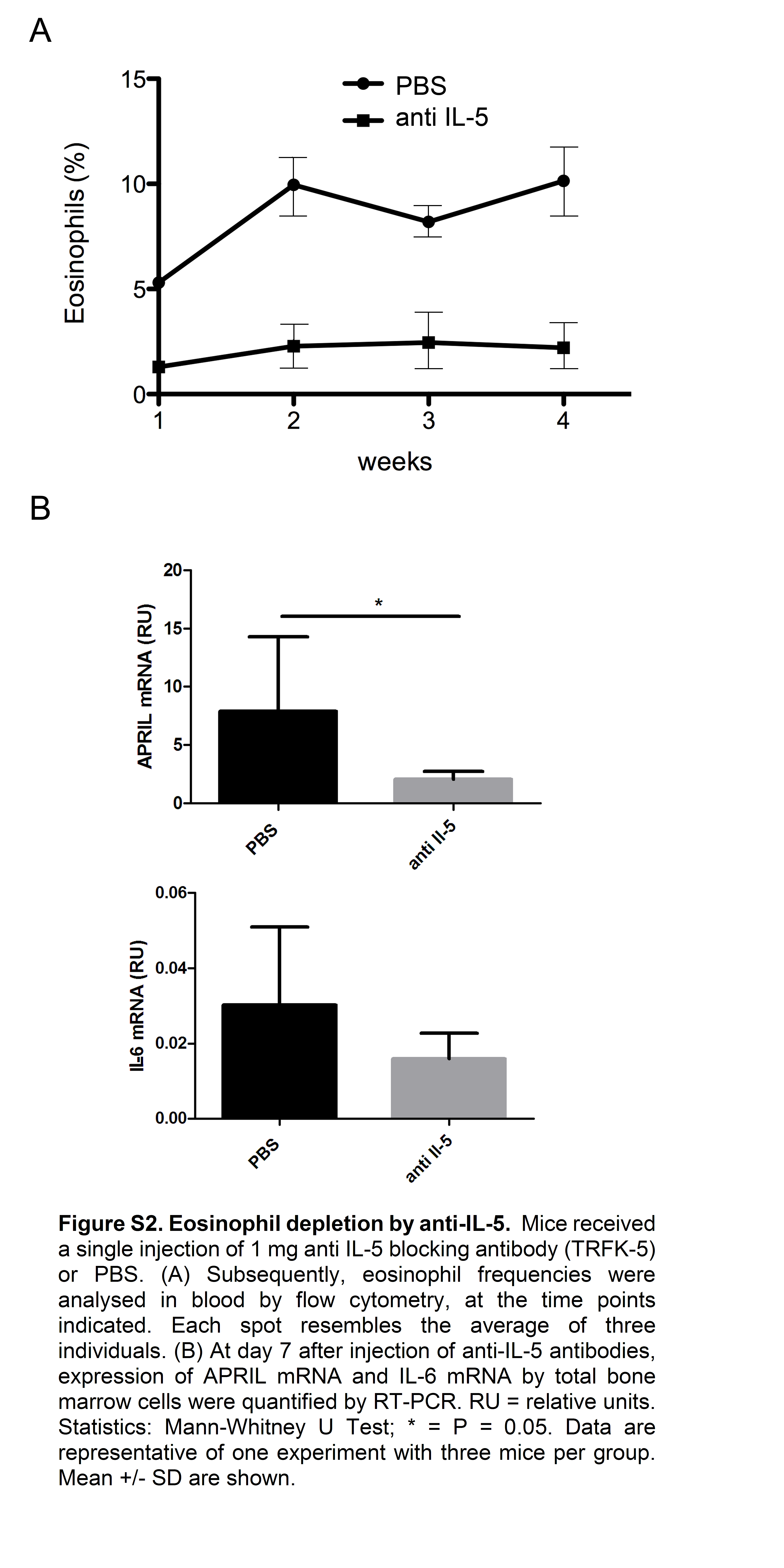

Supplement: Figure S2 — Eosinophil depletion by anti-IL-5. Mice received a single injection of 1 mg anti IL-5 blocking antibody (TRFK-5) or PBS. (A) Subsequently, eosinophil frequencies were analysed in blood by flow cytometry, at the time points indicated. Each spot resembles the average of three individuals. (B) At day seven after injection of anti-IL-5 antibodies, expression of APRIL mRNA and IL-6 mRNA by total bone marrow cells were quantified by RT-PCR. RU = relative units. Statistics: Mann-Whitney U Test; * = P≤0.05. Data are representative of one experiment with three mice per group. Mean +/− SD are shown. (TIF) [file pone.0109018.s002.tif]

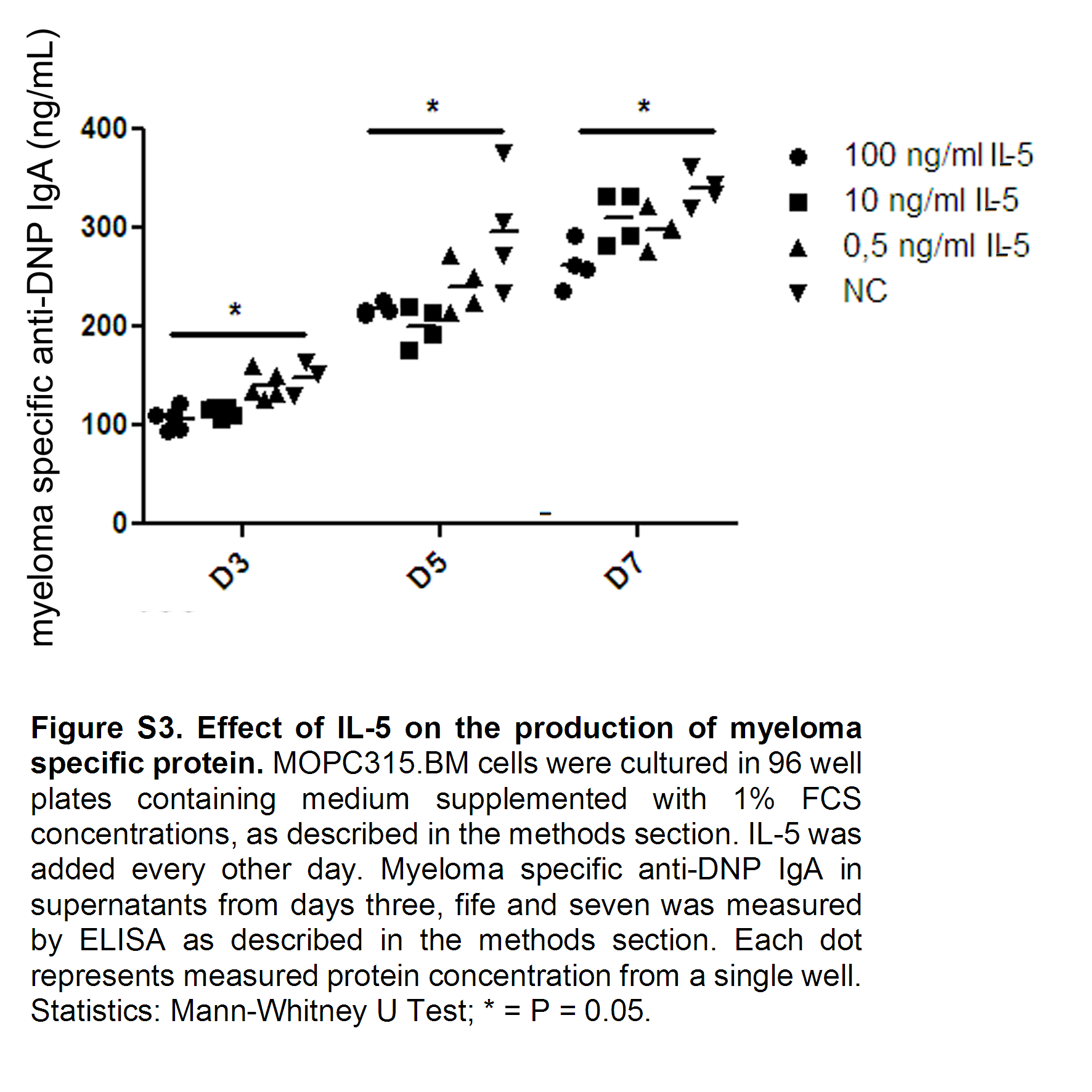

Supplement: Figure S3 — Effect of IL-5 on the production of myeloma specific protein. MOPC315.BM cells were cultured in 96 well plates containing medium supplemented with 1% FCS concentrations, as described in the methods section. IL-5 was added every other day. Myeloma specific anti-DNP IgA in supernatants from days three, fife and seven was measured by ELISA as described in the methods section. Each dot represents a single well. Statistics: Mann-Whitney U Test; * = P≤0.05. (TIF) [file pone.0109018.s003.tif]

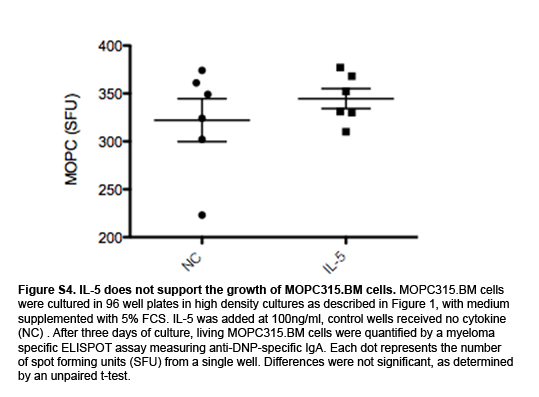

Supplement: Figure S4 — IL-5 does not support the growth of MOPC315.BM cells. MOPC315.BM cells were cultured in 96 well plates in high density cultures as described in Figure 1, with medium supplemented with 5% FCS. IL-5 was added at 100 ng/ml, control wells received no cytokine (NC). After three days of culture, living MOPC315.BM cells were quantified by a myeloma specific ELISPOT assay measuring anti-DNP-specific IgA. Each dot represents the number of spot forming units (SFU) from a single well. Differences were not significant, as determined by an unpaired t-test. (TIF) [file pone.0109018.s004.tif]
